# How the Asthmatic Patients Handle During War?

**DOI:** 10.1002/iid3.70447

**Published:** 2026-04-20

**Authors:** Anna Kovchun, Ayse Füsun Kalpaklioglu

**Affiliations:** ^1^ Sumy State University Sumy Sumy Region Ukraine; ^2^ Kırıkkale Üniversitesi Tip Fakültesi Yahsihan Kırıkkale Türkiye

## Abstract

**Background:**

Psychological stress is increasingly recognized as an important modifier of asthma severity and control. However, objective clinical data on asthma outcomes during prolonged armed conflict remain limited. This study aimed to evaluate changes in anxiety, depression, lung function, and asthma control among patients with persistent asthma living in a frontline region of Ukraine during periods of differing military intensity, and to examine their associations with clinical parameters and access to controller therapy.

**Methods:**

This prospective observational study included 49 adults with persistent asthma and 21 healthy controls examined twice between May–June and September–October 2024 at a regional hospital in Sumy, Ukraine. Anxiety and depression were assessed using the Hospital Anxiety and Depression Scale (HADS). Asthma‐related outcomes included forced expiratory volume in 1 s (FEV₁), Asthma Control Test (ACT) scores, blood eosinophils, and total IgE levels. Statistical analysis was performed using IBM SPSS Statistics 30.0. Pearson correlation analysis was applied to assess relationships between psychological and clinical variables. Binary logistic regression was used to identify factors associated with poor asthma control (ACT < 20), with anxiety and depression entered as continuous predictors and access to baseline therapy as a binary variable.

**Results:**

Psychological distress increased significantly in both asthma patients and healthy individuals during the period of intensified hostilities. In patients with asthma, worsening security conditions were accompanied by significant declines in FEV₁ and ACT scores (*p* < 0.001), whereas eosinophil counts and total IgE levels remained unchanged. Anxiety was negatively correlated with both FEV₁ and ACT across study periods, while depression showed no significant correlations with clinical indices. Logistic regression revealed that higher anxiety levels were independently associated with poor asthma control in May–June (OR = 1.563, 95% CI 1.012–2.412) and September–October 2024 (OR = 1.539, 95% CI 1.032–2.294). Depression was not significantly related to asthma control at either time point. Limited access to baseline therapy in September–October was associated with increased odds of poor asthma control, but this did not reach statistical significance (OR = 2.889, 95% CI 0.60–13.83).

**Conclusion:**

Prolonged exposure to wartime stress was associated with progressive psychological deterioration and worsening asthma control. Anxiety – already evident after 2 years of conflict – emerged as a consistent determinant of impaired asthma outcomes, whereas depressive symptoms showed no independent association. These findings highlight the importance of integrating mental health screening into asthma management strategies in conflict‐affected regions.

## Graphical abstract

We conducted a study of the features of the clinical course of asthma and the severity of psychological manifestations (Hospital Anxiety and Depression Scale) in asthma patients living in the Sumy region, Ukraine, during different periods of the war in Ukraine. In particular, we compared two periods – May/June 2024 and September/October 2024, because these periods had significant differences in the activity of hostilities.

We found a significant deterioration in the level of anxiety and depression against the background of an increase in the number of artillery shelling, air strikes, and the total duration of air raids in the Sumy region of Ukraine, which is further associated with worsening asthma control and a decrease in FEV_1_ level. Therefore, anxiety has a more significant impact on the clinical course of asthma compared to depression.

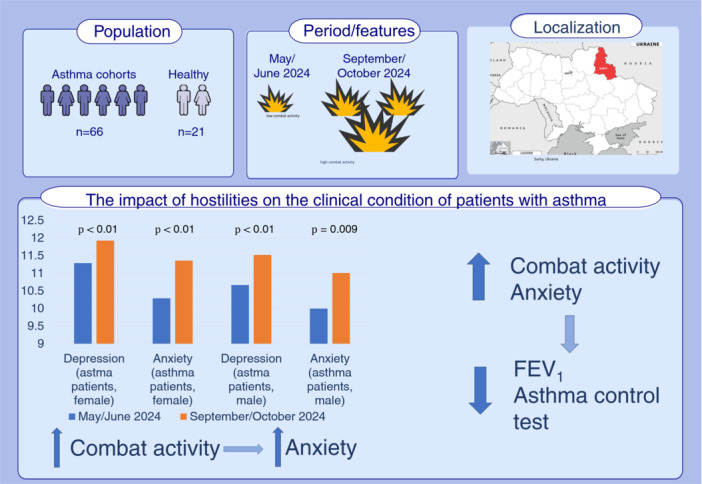

## 1 Introduction

Asthma is a chronic inflammatory airway disease characterized by variable airflow limitation and bronchial hyperresponsiveness, affecting hundreds of millions of individuals worldwide. Its global burden continues to rise, posing substantial challenges to healthcare systems. Beyond its well‐established immunopathological mechanisms, accumulating evidence indicates that psychosocial factors – particularly psychological stress – play a critical role in triggering exacerbations and impairing disease control. A recent study in which the patients were examined at 5‐year intervals reported that the impact of violence throughout life on young patients aged 9–20 years was associated with Th2‐associated asthma, in contrast with the patient with no exposure to violence. These patients had high levels of total IgE or eosinophils, and sensitization to at least one aeroallergen, as well. It has been proven that distress associated with violence leads to a lower response to bronchodilators [1].

The impact of stress and/or any exposure to violence on asthma may depend on heredity, sex or gender, racial or ethnic origin, socioeconomic status, pollutants, comorbidities, and social support. It is proven that chronic distress from prolonged exposure to violence can enhance Th2‐associated responses and accordingly allergic inflammation of the respiratory tract [2, 3, 4, 5].

It has been shown that distress associated with violence can also affect the course of asthma, particularly through neurohormonal pathways. This leads to abnormal gene expression, which regulates the hypothalamic‐pituitary‐adrenal axis, the sympathetic–adrenal–medullary system, and immune responses, either directly or through epigenetic mechanisms such as DNA methylation [6, 7, 8]. Moreover, decreased expression of genes that affect BDR and corticosteroid response, including nuclear receptor subfamily 3, member 1 group C, ADRB2, and corticotropin‐releasing hormone receptor 1 [6, 9], were also reported.

Most studies on the association between stress and asthma involve young patients or children. However, Han et al. studied 81,105 Britons over 40 years of age and found that any previous childhood maltreatment was associated with a 22% increase in the likelihood of asthma in adulthood compared with no episodes of abuse [10]. In a follow‐up study by the same authors, having at least two types of childhood maltreatment was associated with a 1.25–1.59‐fold increased chance of asthma irrespective of sex or eosinophil levels [11]. A systematic review demonstrated that posttraumatic stress disorder (PTSD) is present in 5%–24% of individuals affected by natural disasters [14]. However, the objective assessment of the links between asthma and natural disasters is a complex task, as is isolating the impact of combat actions on asthma. This is explained by the fact that, in addition to stress, such cases involve increased exposure to allergens such as mold, pollen, air pollution, and also create conditions in which most patients have limited access to medical care [15, 16].

The results of a study consisted of 11,481 World Trade Center rescuers: probable PTSD was associated with less bronchodilator response that is a predictor of asthma attack among all participants (adjusted odds ratio = 1.43; 95% confidence interval = 1.19–1.72) [12].

The association between stress and asthma is complex and multifaceted. Stress not only exacerbates asthma symptoms but also influences medication adherence and overall quality of life in individuals with asthma.

But our knowledge about asthma and war association is scares. War and asthma intersect in various ways, particularly regarding the impact of conflict on health, environmental conditions, and access to care. Violence involving the use of firearms throughout life (defined as hearing gunfire more than once) was found to be associated with a 1.8 times higher likelihood of physician diagnosed asthma [13].

While the physical symptoms of asthma are well‐documented, the psychological impact of the disease, particularly its association with anxiety, is increasingly recognized. The above‐mentioned facts regarding the impact of war on the course of asthma are mostly theoretical; difficulties in such studies typically arise at the stage of conditional “standardization” of stress.

It should be noted that the intensity of artillery and air strikes in the Sumy region from the beginning of the war until the summer of 2024 fluctuated between periods of relative calm, affecting mainly settlements within a 10‐km zone, and episodes of large‐scale attacks across the entire region involving aerial weapons. Since August 2024, daily strikes on civilian infrastructure – including universities, schools, kindergartens, hospitals, and nursing homes – have become increasingly common, often occurring at night or early in the morning when civilians are resting and when patients and healthcare workers are arriving at medical facilities. Such living conditions are physically and psychologically exhausting, as the constant threat to safety contributes to anxiety disorders and deterioration in quality of life.

**Figure 1:**
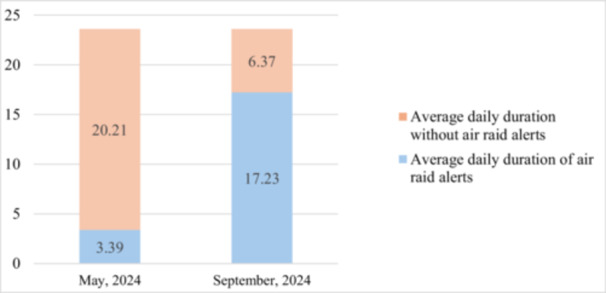
Average daily duration of air raid alerts in the Sumy region of Ukraine in May and September 2024.

**Figure 2:**
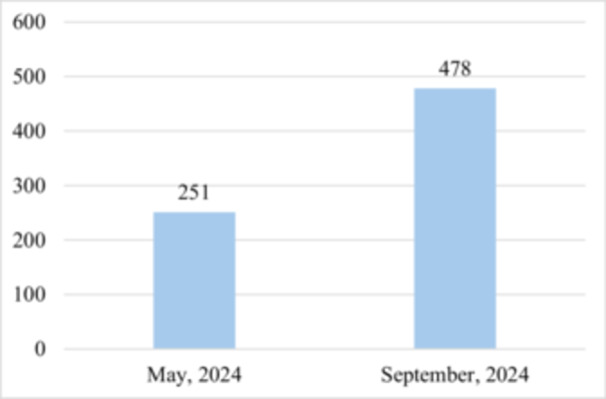
The number of recorded artillery attacks of Sumy region (Ukraine) in May and September 2024.

Concurrently, the frequency and duration of air raid alerts increased sharply. For comparison, in May 2024, the average daily duration of air raid alerts in Sumy was 3 h and 39 min, whereas in September it rose to 17 h and 23 min (Figure 1). Artillery attacks in the Sumy region were recorded 251 times in May and 478 times in September (Figure 2). Given the extremely limited intervals without air raid alerts during the day, patients were frequently unable to follow safety protocols and seek shelter during every warning. Consequently, prolonged and repeated stays in shelters are unlikely to account for the observed deterioration in asthma control during this period.

Against this background of rapidly evolving military conditions and sustained psychological strain, we designed a prospective observational study to evaluate how fluctuations in the intensity of hostilities influenced psychological well‐being and asthma‐related outcomes in patients living in the Sumy region. Specifically, we aimed to assess changes in anxiety and depressive symptoms, lung function, inflammatory markers, and asthma control during two distinct periods characterized by different levels of military activity, and to examine the associations between psychological factors, clinical parameters, and access to baseline asthma therapy.

## 2 Materials and Methods

This study was conducted at the Sumy Regional Clinical Hospital from May to October 2024, the study was approved by the local bioethics committee. The study included 49 patients with persistent asthma, including 28 F/21 M. The control group consisted of 21 healthy individuals (13 F/8 M), average age of patients 38 ± 3.2 y.o. All patients provided informed consent to participate in this study.

Given the significant deterioration of the security situation in Sumy and the increase in the number and duration of air raid alerts in September–October 2024 compared to May–June 2024, which undoubtedly contributed to psychological stress for all residents of the Sumy region, we decided to investigate how this affected the severity of anxiety and depression in all studied patients. All patients were examined twice with an interval of 4 months – May–June 2024 and September–October 2024. Subjects were assessed for depression and anxiety using the Hospital Anxiety and Depression Scale (HADS), patients with asthma were also assessed for blood eosinophils and total IgE levels, forced expiratory volume in first second (FEV_1_) and asthma control test (ACT). To examine psychological status, all patients were asked to complete the HADS [17]. The HADS consists of 14 questions in which the severity of anxiety and depression is rated on a 4‐point scale. The HADS total score was calculated as the sum of anxiety and depression. HADS is a self‐assessment scale is a reliable instrument developed for detecting states of depression and anxiety in the setting of a hospital medical outpatient clinic. The anxiety and depressive subscales are also valid measures of severity of the emotional disorder. In the present study, anxiety and depression were analyzed separately using the corresponding subscales of the Hospital Anxiety and Depression Scale (HADS‐A and HADS‐D), rather than the total HADS score. This approach allowed us to evaluate the independent associations of anxiety and depressive symptoms with clinical and functional asthma‐related parameters.

Statistical analysis was performed using IBM SPSS Statistics 30.0. Data are presented as mean ± standard error. Between‐group comparisons were performed using independent samples *t*‐test. Within‐group comparisons between two time points were conducted using paired samples *t*‐test. Correlations between psychological scores and clinical parameters were assessed using Pearson's correlation coefficient. Binary logistic regression analysis was used to evaluate factors associated with poor asthma control, defined as an ACT score < 20. Anxiety and depression scores were entered as continuous variables, while accessibility to baseline asthma treatment was included as a binary predictor (yes/no). Results are presented as odds ratios (OR) with 95% confidence intervals. A *p*‐value < 0.05 was considered statistically significant.

## 3 Results

During both study periods, we found that the level of depression and anxiety did not differ between genders (*p* > 0.05) in all groups.

**Figure 3:**
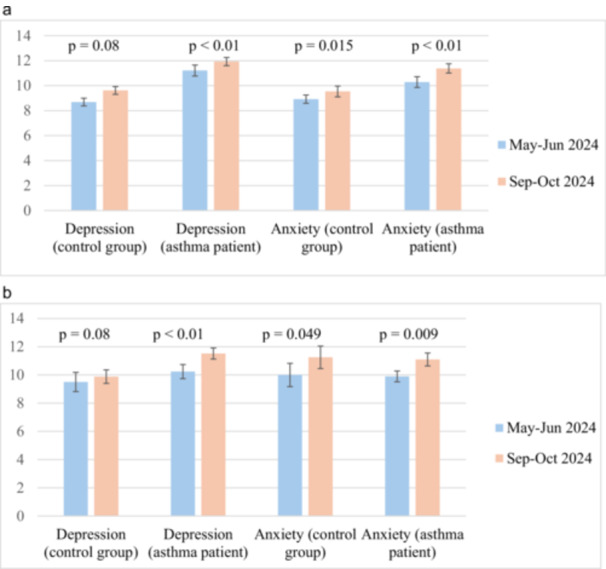
(a) Dynamics of changes in Hospital Anxiety and Depression Scale indicators in females. (b) Dynamics of changes in Hospital Anxiety and Depression Scale indicators in males.

Comparing the severity of depression and anxiety in patients with asthma and in healthy individuals, it was found that depression and anxiety were more pronounced in female asthmatics both during May–June 2024 period (*p* < 0.01, f = 16.3; *p* = 0.048, f = 4.1) and September–October 2024 period (*p* < 0.01, f = 19.1; *p* = 0.006, f = 8.5). However, in males with asthma and in control group, the HADS scores were similar during both study periods (*p* = 0.27, f = 5.44; *p* = 0.863, f = 0.30) (Table 1, Figure 3a,b).

**Table 1:** Significance of differences between the severity of depression/anxiety in patients with asthma and the control group.

Gender	Patient group			Control group		
	F (*n*: 28)	*р* 1	M (*n*: 21)	F (*n*: 13)	*р* 1	M (*n*: 8)
HADS‐D May–June 2024	11.29 ± 0.411	0.295	10.67 ± 0.392	8.69 ± 0.308	0.235	9.5 ± 0.681
	*р*_2 _< 0.001; *р* 3 = 0.137					
HADS‐D September–October 2024	11.93 ± 0.329	0.432	11.52 ± 0.394	9.62 ± 0.311	0.639	9.88 ± 0.479
	*р*_2 _< 0.001; *р*_3_ = 0.27					
HADS‐A May–June 2024	10.29 ± 0.430	0.628	10.00 ± 0.359	8.92 ± 0.329	0.175	10.0 ± 0.824
	*р* 2 = 0.050; *р*_3_ = 1.0					
HADS‐A September–October 2024	11.36 ± 0.372	0.656	11.10 ± 0.457	9.54 ± 0.433	0.053	11.25 ± 0.796
	*р*_2_ = 0.006; *р* 3 = 0.863					

^1^ *p* – significance of differences between the severity of depression/anxiety in male and female.

^2^ *p* – significance of differences between the severity of depression/anxiety in female with asthma and control group.

^3^ *p* – significance of differences between the severity of depression/anxiety in male with asthma and control group.

Additionally, we assessed and compared the HADS total score in May–June 2024 and September–October 2024, but the differences were not clinically significant (*p* = 0.05 and *p* = 0.18).

Figure 3a,b demonstrates significant changes in all groups, indicating an increase in depression and anxiety both in patients with asthma and in healthy individuals against the backdrop of intensified military activity, despite 2 years of full‐scale war.

In our study, we also analyzed the dynamics of changes in FEV_1_ level, ACT result, eosinophils, and total IgE levels (Table 2). It was found that in asthma patients, the escalation of hostilities significantly worsened FEV_1_ levels and ACT results (*p* < 0.001). However, laboratory markers (eosinophil and total IgE levels) didn't show significant changes (*p* = 0.870 and *p* = 0.05).

**Table 2:** Dynamics of changes in key instrumental and laboratory indicators in asthma patients under increasing stress.

Parameter	May–June 2024	September–October 2024
FEV_1_, %	66.06 ± 1.23	62.78 ± 1.21
	*t* = 6.56, *p* < 0.001	
АСТ	17.10 ± 0.52	15.32 ± 0.601
	*t* = 6.36, *p* < 0.001	
Blood eosinophils, %	5.8 ± 0.39	5.14 ± 4.14
	*t* = 2.007, *p* = 0.050	
IgE, IU/mL	305.63 ± 61.4	302.39 ± 50.27
	*t* = 0.165, *p* = 0.870	

Given the significant changes in clinical indicators like FEV_1_ and ACT against the background of deteriorating security conditions, we performed correlation analysis between the level of anxiety and/or depression with these parameters as an indicator of asthma control. As a result, we found a negative significant correlation between the anxiety indicator and FEV_1_ and ACT during both study periods (*r* = −0.573, *p* < 0.001; *r* = −0.432, *p* = 0.002), and *r* = −0.621, *p* < 0.001; *r* = −0.351, *p* = 0.013, respectively), showing that anxiety has a significant impact on the clinical course of asthma. However, the correlations between depression and FEV_1_/ACT were not significant (*p* = 0.94; *p* = 2.17).

Binary logistic regression analysis evaluating the impact of access to baseline asthma therapy on asthma control in May–June 2024 was not performed because all patients had uninterrupted access to regular controller treatment during that period, resulting in a lack of variability in the exposure variable. In September–October 2024, when access to baseline therapy became limited, lack of access to controller inhalers was associated with a higher likelihood of poor asthma control; however, this association did not reach statistical significance (OR = 2.889, 95% CI 0.60–13.83, *p* = 0.184). The absence of a statistically significant relationship may reflect the resilience of healthcare delivery in the region. Despite severe operational challenges, clinicians who remained in their communities and pharmacists who ensured medication supply likely contributed to maintaining continuity of asthma care, while temporary shortages were often mitigated by professional recommendations regarding alternative therapies.

In contrast, psychological factors demonstrated a more consistent relationship with asthma control. In May–June 2024, during a period of relatively lower military activity, higher anxiety levels were significantly associated with poor asthma control, defined as an ACT score < 20 (OR = 1.563, 95% CI 1.012–2.412, *p* = 0.044), whereas depression was not significantly related to asthma control (OR = 1.179, 95% CI 0.854–1.629, *p* = 0.317).

Similarly, in September–October 2024, against the backdrop of intensified hostilities, anxiety remained significantly associated with impaired asthma control (ACT < 20) (OR = 1.539, 95% CI 1.032–2.294, *p* = 0.034), while depressive symptoms again showed no statistically significant association (OR = 1.413, 95% CI 0.903–2.210, *p* = 0.130).

## 4 Conclusion

This study demonstrates that prolonged exposure to wartime stress is associated with a progressive deterioration in psychological well‐being and asthma control among patients living in an active conflict zone. Despite more than 2 years of full‐scale war, individuals had already developed clinically relevant anxiety by May 2024, even during a period of relatively lower military activity, and this anxiety was significantly linked to poorer asthma control. With the subsequent escalation of hostilities in September–October 2024, psychological distress further intensified, accompanied by worsening lung function and asthma control, underscoring the cumulative impact of chronic stress on respiratory disease.

Female patients with asthma exhibited more pronounced symptoms of anxiety and depression compared with healthy women, whereas no significant gender differences were observed within the asthma group itself. Although access to baseline asthma therapy became limited later in the year, the absence of a statistically significant association between medication availability and asthma control likely reflects the remarkable resilience of regional healthcare systems and the adaptive strategies employed by clinicians and pharmacists to maintain continuity of care under extreme conditions.

Across both study periods, anxiety emerged as a more consistent determinant of poor asthma control and airway obstruction than depressive symptoms, highlighting its central role in the clinical course of asthma during sustained psychological adversity. These findings emphasize the necessity of integrating systematic mental health screening – particularly for anxiety – into routine asthma management for populations exposed to prolonged conflict. Multidisciplinary approaches that address both respiratory and psychological dimensions of disease may be essential for improving outcomes and preserving quality of life in such high‐risk settings.

## Author Contributions

**Anna Kovchun:** writing – original draft, visualization, validation, software, investigation, methodology, writing – review and editing, conceptualization, data curation, resources, formal analysis, project administration. **Ayse Füsun Kalpaklioglu:** writing – review and editing, conceptualization, formal analysis, project administration, supervision.

## Conflicts of Interest

The authors declare no conflicts of interest.
